# Virtual excavation and analysis of the early Neanderthal cranium from Altamura (Italy)

**DOI:** 10.1038/s42003-023-04644-1

**Published:** 2023-03-24

**Authors:** Antonio Profico, Costantino Buzi, Fabio Di Vincenzo, Marco Boggioni, Andrea Borsato, Giovanni Boschian, Damiano Marchi, Mario Micheli, Jacopo Moggi Cecchi, Marco Samadelli, Mary Anne Tafuri, Juan Luis Arsuaga, Giorgio Manzi

**Affiliations:** 1 Department of Environmental Biology, Sapienza University of Rome, Rome, 00185 Italy; 2 Department of Biology, University of Pisa, Pisa, 56126 Italy; 3 Catalan Institute of Human Paleoecology and Social Evolution (IPHES-CERCA), Tarragona, 43005 Spain; 4 Àrea de Prehistòria, Facultat de Lletres, Universitat Rovira i Virgili, Tarragona, 43005 Spain; 5 Natural History Museum – Palazzo Nonfinito, University of Florence, Florence, 50122 Italy; 6 School of Paleoanthropology, University of Perugia, Perugia, 06123 Italy; 7 School of Environmental and Life Sciences, The University of Newcastle, Callaghan, NSW 2308 Australia; 8 Palaeo-Research Institute, University of Johannesburg, P.O. Box 524, Johannesburg - Auckland Park, 2006 South Africa; 9 Centre for the Exploration of the Deep Human Journey, University of the Witwatersrand, Private Bag 3, Wits, Johannesburg, 2050 South Africa; 10 Department of Humanities, Roma Tre University, Rome, 00185 Italy; 11 Department of Biology, University of Florence, Florence, 50122 Italy; 12 Institute for Mummy Studies, EURAC Research, Bolzano, 39100 Italy; 13 Centro Mixto UCM-ISCIII de Evolución y Comportamiento Humanos, Madrid, 28029 Spain; 14 Departamento de Paleontología, Facultad Ciencias Geológicas, Universidad Complutense de Madrid, Madrid, 28040 Spain

**Keywords:** Evolution, Palaeontology

## Abstract

Complete Neanderthal skeletons are almost unique findings. A very well-preserved specimen of this kind was discovered in 1993 in the deepest recesses of a karstic system near the town of Altamura in Southern Italy. We present here a detailed description of the cranium, after we virtually extracted it from the surrounding stalagmites and stalactites. The morphology of the Altamura cranium fits within the Neanderthal variability, though it retains features occurring in more archaic European samples. Some of these features were never observed in *Homo neanderthalensis*, i.e. in fossil specimens dated between 300 and 40 ka. Considering the U-Th age we previously obtained (>130 ka), the morphology of Altamura suggests that the archaic traits it retains may have been originated by geographic isolation of the early Neanderthal populations from Southern Italy.

## Abstract (web summary)

3D reconstruction of a human cranium held within a deep cave in Italy is achieved with advanced virtual anthropology techniques; its features indicate that geographic isolation contributed to variation and evolution along the Neanderthal lineage.

## Introduction

Several models have been proposed to explain the evolution of the Neanderthal lineage echoed in the phenotype of available European fossils of Middle (Chibanian) and early Late Pleistocene^1–6^. On-going debate about these models involves integration, mutual influence, adaptive significance, and stochastic occurrence of Neanderthal features.

Looking at the fossil record, we can discern an evolutionary transition that corresponds to Marine Isotope Stages (MIS) from 7 to 5^7^; in these “early” Neanderthals (ERN), the typical morphological traits are absent or weakly expressed while the extreme degree of encephalization—common among the so-called “classic” Neanderthals, or EWN (European Würmian Neanderthals), corresponding to MIS 4–3—had yet to be fully reached^8^. Nevertheless, there is consensus on ascribing both the ERN and the EWN samples to *Homo neanderthalensis*, as various archaeological, morphological, and paleogenetic indicators concurrently suggest^2^.

**Fig. 1:**
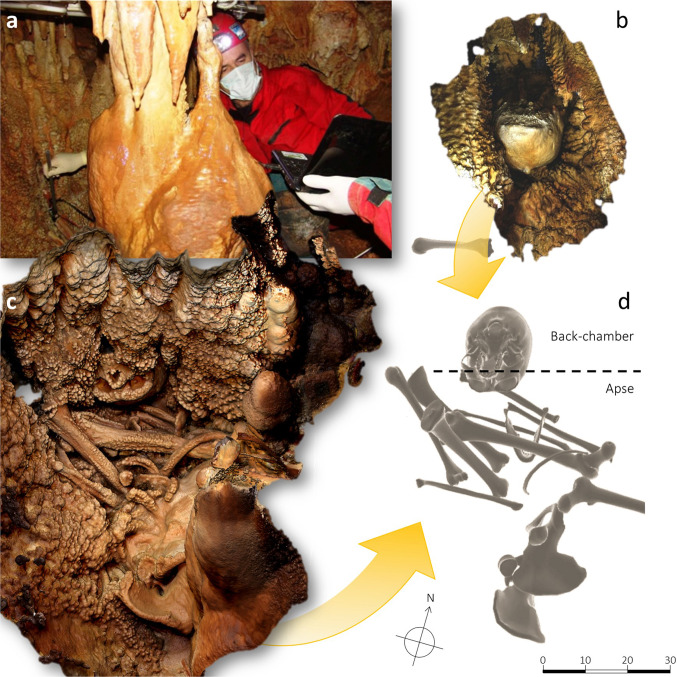
The Altamura cranium straddles two chambers within the karstic system. Photographic probes (**a**) were used to acquire photogrammetrically the basal and posterior components (BP) of the cranium (**b**), exposed in the Back-chamber. Its frontal and facial parts (FF) were acquired by laser scanner from the Apse (**c**), where also the mandible and several long bones are directly visible on the cave floor (**c**, **d**).

The specimen found in 1993 in a cave near the town of Altamura, in Southern Italy, is the most intriguing example among the ERN variability^9–12^ (Supplementary Data Figs. 1, 2 and ‘Methods’). Altamura represents a unique example of potentially complete non-modern human skeleton^12–14^ (Fig. 1) that may shed new light on a poorly known phase of Neanderthal evolution. Still, it waits to be extracted from the cave in order to be comprehensively described. Studies carried out in the last decade ascribed Altamura to *Homo neanderthalensis*, following morphometric and paleogenetic (mtDNA) data associated to a U/Th age ranging between 130.1 ± 1.9 and 172 ± 15 ka^9^. The skeleton is disarticulated and encrusted with calcite coatings, whose early formation favoured the exceptional preservation of even its most fragile skeletal structures^13^. Considering these extraordinary conditions, carefully planned in situ studies are necessary before a permit to physically extract the various bones can be issued (Supplementary Data Fig. 3).

We present here—for the first time—the morphology of the Altamura cranium. We virtually reconstructed the cranium by acquiring its shape in two separate parts, which we subsequently re-assembled using a protocol designed for this purpose^15^. After this procedure, we describe and contextualise its overall external morphology within the European human variability of the Middle and early Late Pleistocene.

## Specimen context

The skeleton lies in a small chamber—the so-called “Apse of the Man” (hereafter Apse)—situated to the north-western end of the Lamalunga karst system, which can be accessed only by speleological techniques. Most—probably all—the skeletal elements collapsed here after the death of the individual and the decay of the soft tissues (Supplementary Data Fig. 2). Beyond the Apse there is the “Back-chamber”, a narrow cavity separated from the Apse by a curtain of columnar speleothems (Fig. 1a–d). The inside of this chamber— containing a few human bones belonging to the same skeleton—can only be observed with difficulty through a few narrow gaps in the speleothem curtain.

The cranium is cemented upside down within the speleothem curtain, with stalagmites adhering to its sides; its anterior part faces the Apse, whereas the rear and the base face the Back-chamber. Consequently, half the cranium (face and frontal bone, FF) is visible from the Apse, whereas the other half (palate, cranial base and most of the cranial vault, BP) is accessible only via indirect inspection with probes through the gaps in the speleothem curtain.

Most of the cranium is coated with speleothem crusts; mm- to cm-thick coralloid concretions cover the anterior parts, mostly the projecting ones^13^, whereas a uniformly thin calcite layer coats the rear and the base, including the whole occipital bone where the anatomical structures are consequently more clearly visible.

## Results

### Virtual model

**Fig. 2:**
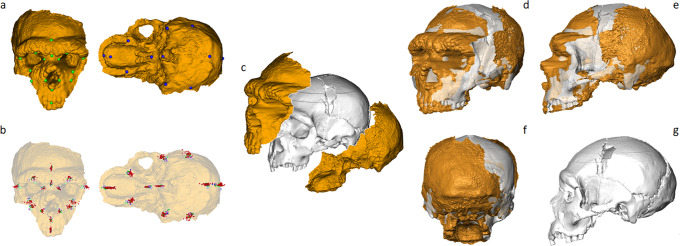
Virtual reconstruction of the Altamura cranium. **a** Homologous landmark configurations were acquired on the FF and BP of Altamura and of a reference specimen, the nearly complete Cranium 5 from the Sima de los Huesos site (SH-5); **b** landmark coordinates were processed separately by Generalised Procrustes Analysis (GPA); **c** FF and BP were aligned according to the reference specimen showing the highest morphological affinity; **d**–**f** various views of the virtual model of Altamura aligned on SH-5; **g** SH-5 lateral view.

Digitally acquiring this specimen was particularly challenging because of the very peculiar setting of the remains. The two exposed parts of the cranium had to be acquired separately, the back one by probes, without seeing it directly and working in an uncomfortable environment. Consequently, we acquired separately the FF and BP parts (Fig. 2). The resulting digitised parts do not have conjoining points, so that a new method had to be developed to re-assemble them. We therefore decided to virtually combine the two halves as if they were disarticulated portions of a cranium, by using reference specimens as templates in finding the best fit.

The reference sample (*N* = 37) we used includes modern humans, Neanderthals (ERN and EWN) and other representative European Middle Pleistocene humans (MPH) (Supplementary Data Table 1). The morphology of each specimen was coded by means of geometric morphometrics, acquiring 16 anatomical points on FF and 12 on BP (Fig. 2a, b and Supplementary Data Table 2).

We measured the morphological distance (Euclidean and Procrustes distances) of both the Altamura FF and BP anatomical points from the homologous ones of each specimen in the reference sample (Supplementary Data Fig. 2 and ‘Methods’). Cranium 5 from Atapuerca - Sima de los Huesos^6,16^ (SH-5, Fig. 2g) showed the highest morphological affinity with Altamura, followed by La Chapelle, La Ferrassie and Guattari (EWN) and Saccopastore 1 (ERN) (Supplementary Data Figs. 4, 5). SH-5 was consequently used as the best reference to refit the cranial components of Altamura by using a computer-assisted semi-automatic protocol^15^ (Fig. 2 and ‘Methods’).

Following this virtual reconstruction, the estimated endocranial volume of Altamura is 1190 cc (see Methods, ‘Estimation of cranial capacity’), close to other ERNs as Apidima 2 (1290 cc)^17^, Krapina (*N* = 5, 1306.40 ± 105.49 cc)^18^, Saccopastore 1 (1174 cc)^19^. The average endocranial capacity in EWNs is 1463.13 ± 168.02 cc (*N* = 16) (Supplementary Data Table 3). Therefore, after the reconstruction of the entire cranium, the endocast of Altamura (Supplementary Data Fig. 6), is quantitatively closer to “early” (ERN) than to “classic” (EWN) Neanderthals: this result is definitely consistent with its chronology, which clearly predates the Late Pleistocene.

It should be noted that the morphological affinity between Altamura and SH-5 is also reflected in some archaic discrete traits shared by the two specimens, despite their different chronology (Fig. 2d–g). These features include relevant aspects: namely, the angled coronal profile—i.e., absent or moderate lateral inflation of the parietals, unlike the typical *en-bombe* morphology of *Homo neanderthalensis*—and the protruding mastoid processes (Supplementary Data Fig. 7). Even the facial morphologies of Altamura and SH-5 show similar receding zygomatic arches and frontal squama, whereas they differ in the morphology of the supraorbital arches. In general, the cranial architecture of Altamura looks like an antero-posteriorly “stretched” variant of SH-5, with anterior projection of the mid-face, lower cranial vault, different orientation of the mastoids and different shape of the browridges.

**Fig. 3:**
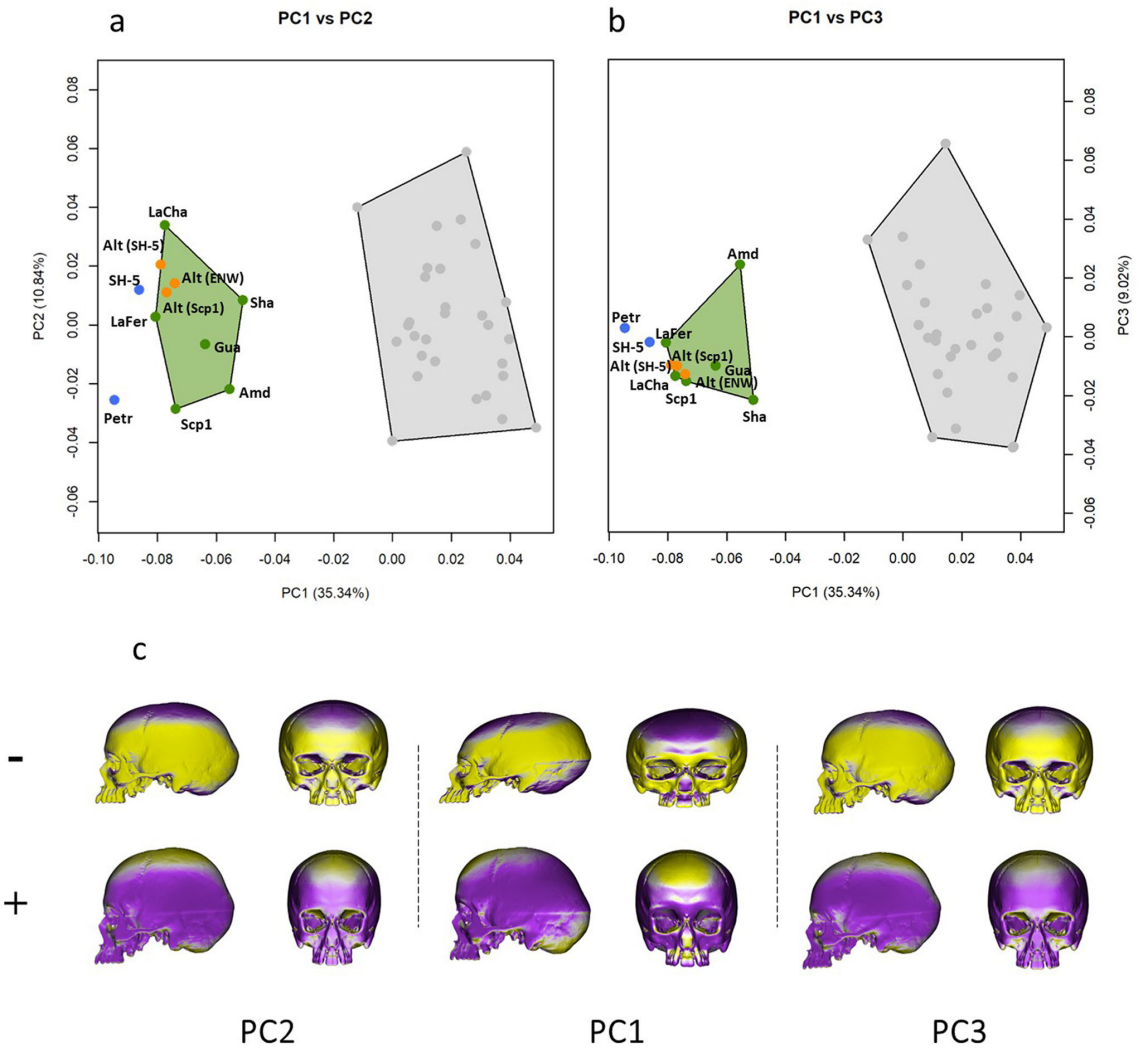
Principal Component Analysis comparing Altamura with crania of Neanderthal lineage and of modern humans. **a** PC1 vs PC2. **b** PC1 vs. PC3. Full circles represent fossil specimens; orange: virtual reconstructions of Altamura based on SH-5 (Alt-SH-5), on the mean shape of the EWN sample (Alt-ENW) and on Saccopastore 1 (Alt-Scp1); blue: Mid-Pleistocene specimens (SH-5: Sima de los Huesos 5; Petr: Petralona); green: *Homo neanderthalensis* (Amd: Amud; Gua: Guattari 1; LaFer: La Ferrassie; LaCha: La Chapelle; Scp1: Saccopastore 1; Sha: Shanidar); grey: *Homo sapiens*. **c** Cranial morphology associated at extreme negative (−) and positive (+) values of the first three principal components; yellow and violet respectively indicate local expansion and contraction from the mean shape.

Furthermore, three possible models of the Altamura cranium—resulting from virtual alignments with different templates such as SH5, Saccopastore 1 and the EWN consensus—were compared with Neanderthals and modern humans, using PCA on the full cranial landmark configuration (Fig. 3 and ‘Methods’). PC1 (35.34% of the total variance) discriminates between modern and extinct humans, with MPH, ERN and EWN grouping along negative values, and modern humans on the positive ones—characterised by smaller face, bulged frontal bone, globular and antero-posteriorly shorter neurocranium. Notably, PC1 clusters the Altamura models within the Neanderthal variability (Fig. 3a); they also fit within the Neanderthal variability of PC2-3 (10.84% and 9.02%) (Fig. 3b), showing midfacial prognathism and limited basicranial flexion, though their neurocranial height and antero-posterior length are rather reduced (Fig. 3c).

### Discrete features

We examined 20 derived Neanderthal traits^4^ to supplement information to be used in assessing the phylogenetic position of Altamura (‘Methods’ and Supplementary Data Table 4).

Neanderthal features are largely predominant in Altamura (15/20), more than in the Atapuerca sample SH (*N* = 17; 10/20) and less than in the Saccopastore specimens (*N* = 2; 17/20) (Supplementary Data Fig. 8). Altamura also shows affinities with other MPH specimens regarding several diagnostic features. The parietals are angulated along the coronal profile (i.e., absence of the *en-bombe* shape in posterior view), approaching the Atapuerca SH sample and Petralona, whereas those of the Neanderthals (including Saccopastore) are inflated. This shape is consistent with the position of the maximum cranial breadth (144.5 mm), which is close to values occurring among ERN specimens but located at the level of the supramastoid crests. Mastoid tips extending well below the occipitomastoid crest are another archaic feature; notably, elongated mastoids, inclined medially as in Altamura, occur also in Saccopastore^4^.

The shape of the supraorbital torus—which can be inferred, despite the browridge being covered by coralloid concretions—includes features that can be considered as plesiomorphic. This pattern recalls the clear distinction (torsion) between the supraciliary (lateral) and supraorbital (medial) arches, which is observed in European MPH specimens like Arago XXI and Ceprano, or in African specimens like Kabwe (Broken Hill)^20^. This morphology does not occur among the Neanderthals (both ERN and EWN), while the Atapuerca SH sample is characterised by intermediate or variable shapes^6^, being in general more Neanderthal-like (Supplementary Data Fig. 9).

Parsimony strict consensus (Supplementary Data Fig. 8B) and neighbour-joining (Supplementary Data Fig. 8C) clustering methods performed on the entire set of the discrete features show that Altamura is situated between the SH sample and the Neanderthals. The position of the Saccopastore crania within the same diagram is particularly interesting, because these two specimens from Rome are penecontemporaneous with Altamura or even older^21^, but show a closer affinity with the Neanderthal morphology when discrete apomorphic traits are considered.

### The occipital bone

The *planum occipitale* is convex, with extended lambdoidal flattening and a peculiar occipital bun resembling the Neanderthal morphology. There is a single wormian bone close to the left asterion. The angle between the lambda and the asteria is 83.5°, close to values occurring among Europeans of the Middle and early Late Pleistocene (Supplementary Data Fig. 10).

Altamura shows a well-developed and bipartite occipital torus resembling the typical Neanderthal morphology, as it is limited to the medial portion of the squama and tapers out toward the asteria (Supplementary Data Fig. 11). Directly above the superior nuchal line, the supra-iniac fossa is wide although not clearly defined. There is no occipital protuberance, the external occipital crest is marked only at the level of the crucial eminence^22,23^; the Waldeyer’s crests are not conspicuous and a well-developed supramastoid crest can be observed on the left side of the cranium.

**Fig. 4:**
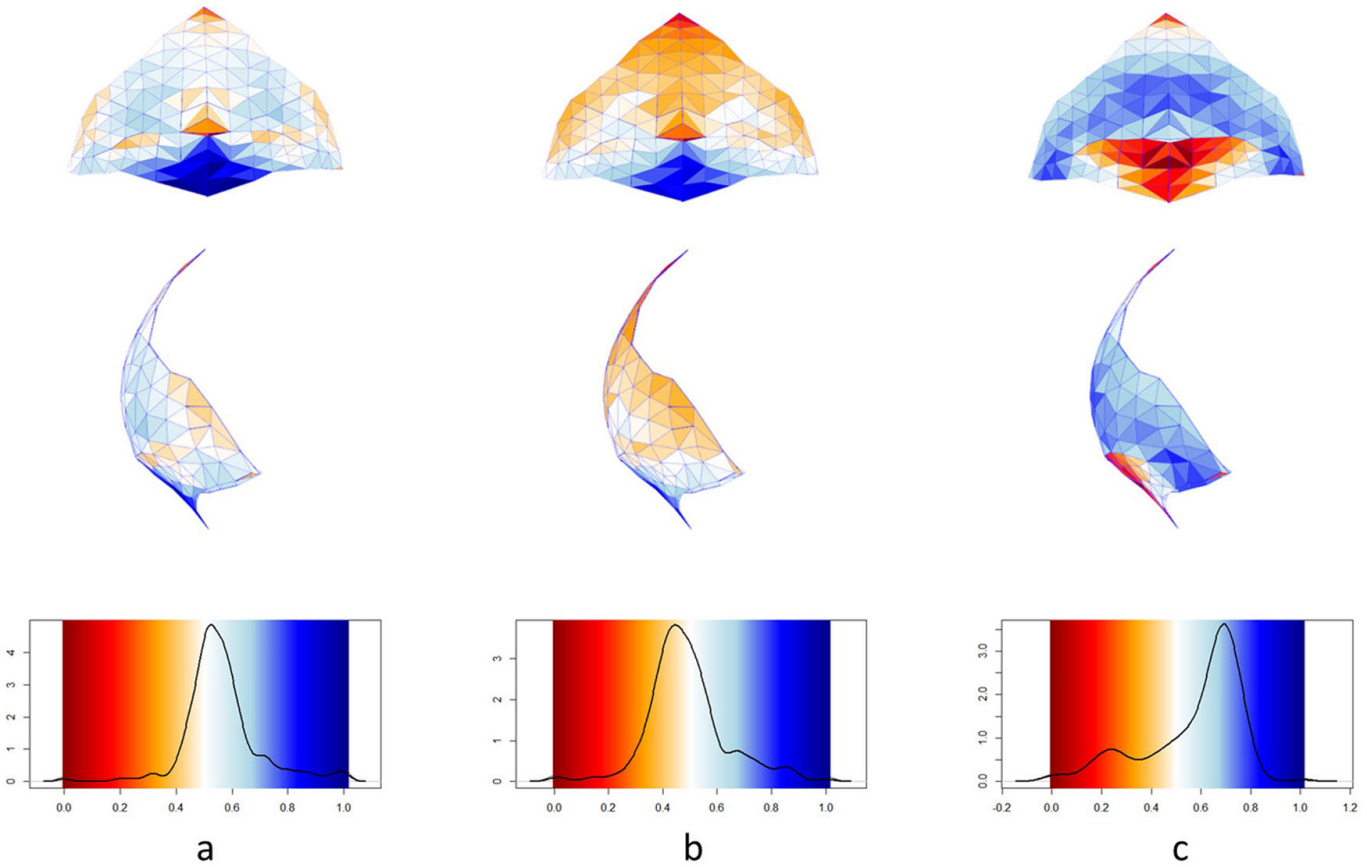
Shape analysis of the occipital squama. 3D map variations of the occipital squama showing Altamura compared to the average morphology of *Homo neanderthalensis* (**a**), Mid-Pleistocene humans (**b**) and *Homo sapiens* (**c**). Colour maps are reported in posterior and lateral view. Warm and cold colours indicate respectively which regions in the three average morphologies are respectively locally contracted and expanded compared to Altamura.

In the PCA analysis of the occipital squama (Fig. 4; Supplementary Data Figs. 12–13 and ‘Methods’), Altamura lies on intermediate values of the PC1 axis (42.7% of the variance), outside the negative values of plesiomorphic, angulated profile shapes. Conversely, it overlaps with derived morphologies associated with a more rounded lateral profile, like modern humans. It also clusters with Neanderthals along the second and third components. However, Altamura differentiates from the EWN sample because of its less typical occipital bun, as highlighted by another PCA performed only on the mid-sagittal profile of the squama (Supplementary Data Fig. 14).

## Discussion

The evolution of the Neanderthals is deeply rooted in the Middle Pleistocene of Europe^1,2^, but the identification of *Homo neanderthalensis* as a species is still far to be clear^3^, particularly with respect to date of its origin. Skeletal morphology is crucial in this respect; the Neanderthal cranium, in particular, is well characterised by a set of evolving features, including: (i) a high endocranial volume, (ii) an elongated antero-posterior cranial vault (shared with other archaic humans), (iii) distinguishing traits, such as the *en-bombe* shape in posterior view, the presence of an occipital chignon, or the mid-facial prognathism^2,4^. Expression of these features, as well as the trend of their occurrence through the Middle and early Late Pleistocene, have been used to assess the origin of the species and to suggest different patterns of evolution^1,5^.

Among these scenarios, the so-called “*accretion model*”^1,2^ argues that the European populations of the Middle Pleistocene evolved in partial genetic isolation from other contemporary African and eastern Eurasian groups since around 500 ka. In this perspective, it is assumed that while morphological traits emerged through time, the phenotypic and genetic variability between and among populations decreased^24–27^. Nevertheless, after a long-lasting debate^1,2,5,6^, there is no unanimous agreement on this scenario^3,28,29^ and exceptions to the accretion process have been pointed out^3,5,28,29^. In this framework, we believe that skeleton from Altamura, particularly its cranium, may shed light on the origin and evolutionary pattern of the Neanderthals.

Altamura has been U/Th dated at more than 130 ka and probably at no more than 172 ka^9^. A first, while accurate, sequences of U/Th samplings of karst concretions accumulated on a bone fragment and on a stalactite close to the skeleton provide, on the one hand, a minimum date (*terminus ante quem*) of 130.1 ± 1.9 and exclude, on the other, a dating in the range between 189 ± 29 and 172 ± 15 ka (warm MIS 7.1)^9^, suggesting that Neanderthal populations lived in southern Italy during MIS 6, a time span characterized by cold temperatures.

Given this chronology, the cranial shape of Altamura is expected to fall within the Neanderthal variability at large, with features shared with the so-called “classic” Neanderthals (EWN group) as well as with the penecontemporaneous Saccopastore 1 (ERN group), as we have shown here and consistently with the previously reported mitochondrial DNA data^9^ and postcranial morphology (right scapula)^9,14^.

By contrast, the similarity with the SH-5 cranium from Sima de los Huesos (ca. 430 ka^6^, MPH group) is rather unexpected. We observed this affinity in the expression of various cranial discrete features, as well as in the overall morphology of the occipital bone. In addition, the midsagittal profile of the occipital in Altamura and its small endocranial volume are more strictly related to the MPH sample than to ERN and EWN groups. The Italian specimen retains also plesiomorphic features in other anatomical regions, including the profile of the cranial vault in posterior view, the prominent mastoids and the shape of the supraorbital torus, whose morphology is apparently close to Arago XXI and Ceprano^30,31^, which do not seem belong to the “Neanderthal lineage”. Under this perspective, the relatively small endocranial capacity is not at all the unique trait differentiating Altamura from the ERN and EWN samples.

Thus, the cranial morphology of Altamura is consistent with the progressive accumulation of Neanderthal traits claimed by the “*accretion model*”^1,2^. At the same time, however, the retention of archaic features (unexpected for its chronology) does not fit a rigid view of such an evolutionary model. Altamura my thus represent the remnant of an archaic population, which was probably not in simple continuity with the Neanderthal lineage, as suggested also by previous studies on ancient mitochondrial DNA and on the functional morphology of the scapula^9,14^. The same issue is raised by the revised age of some European fossils of the Middle Pleistocene^32,33^, suggesting that examples of variable degrees of similarity (or diversity) with the Neanderthal morphology existed simultaneously in separated regions.

As a matter of fact, we may assume that human populations of the Paleolithic as those of the Neanderthal ancestors were fragmented over a wide geographical range; thus, it is reasonable that their characteristics differed from one region to another. A variety of environmental and biological components—including climatic instability^24^, habitat fragmentation^27^, geographical isolation and reduced gene flow^25,26^—were probably crucial factors in shaping the suite of Neanderthal morphological traits, acting in combination as driving forces of human evolution in Europe during the Middle Pleistocene. To make matters more complex, the combination of geographic isolation with a progressive anagenetic lineage may also explain the apparent contrast between a symplesiomorphic (i.e., Denisova-like) mitochondrial DNA and a more derived (Neanderthal-like) nuclear DNA, as it has emerged from the paleogenetic analysis of the fossil material dated to over 430 ka from Atapuerca-Sima de los Huesos, in Spain^34–36^.

In this scenario, Altamura further demonstrates that the southernmost regions of the Italian peninsula, like other regions in Europe^37^, played the crucial role of ecological refugia where plesiomorphic morphologies were preserved for longer times than elsewhere^38–41^. This hypothesis has already been proposed to explain the retention of archaic features in the Middle Pleistocene calvarium from Ceprano, in south-central Italy^33,36^. Therefore, it seems to us that the “accretion model” explains Neanderthal evolution only when a non-linear and ecologically-driven perspective is considered, taking into account the geographic distribution of the fossil record, habitat fragmentation, environmental isolation and local extinctions.

## Methods

### Virtual extraction

We used two different methodologies to digitally acquire the shape of the cranium of Altamura. The front side (FF), which is visible and accessible from the Apse, was acquired via laser scanning with a Konica Minolta range7 at a resolution of 40 μm. The back portion (BP) was acquired via photogrammetry by a GoPro camera mounted on a handheld probe. The GoPro images were subsequently processed by Agisoft Photoscan software. Further observations of the diagnostic traits of the cranium, not aimed at the reconstruction, were carried out by Dino-Lite Digital Microscope AM4113T (Supplementary Data Fig. 3).

### Digital alignment of the Altamura cranium

The two digitised portions were treated as parts of a fragmented skull to be digitally restored. A recently published method designed and coded in R environment was used for this purpose (Digital Tool for Alignment, DTA^15^). DTA finds the best fit in aligning two disarticulated models by using a comparative sample as reference. This method includes the following steps: (i) symmetrisation of the landmark configurations, (ii) scaling of the reference configuration to the size of the target set, (iii) Procrustes registration (i.e., generalized Procrustes analysis) between the scaled reference and the target configuration, (iv) calculation of the cumulative morphological distance between the two aligned configurations (i.e., Euclidean distance). Eventually, the comparative specimen with greatest morphological affinity to Altamura was used as reference to align (translation and rotation) the two disarticulated cranial fragments.

The reference sample we selected comprises digital models of 37 specimens including *Homo sapiens* (*N* = 29), *Homo neanderthalensis* (*N* = 6) and Middle Pleistocene humans (*N* = 2) (Supplementary Data Table 1). We acquired homologous landmark configurations on the FF and BP of each model, consisting of 16 and 12 landmarks respectively (Fig. 2a, b and Supplementary Data Table 2). We chose only anatomical points with no concretions and/or easily recognisable under thin calcite coatings. For the purpose of this analysis (Fig. 2c), the bregma and the two poria were estimated in Altamura by using the Thin-Plate Spline interpolation function^42^, since these landmarks are not clearly visible.

We performed three virtual reconstructions of Altamura using for reference the models of SH-5, Saccopastore 1 and the mean of EWN (Fig. 3 and Supplementary Data Fig. 5). We assessed the phenetic affinity of Altamura among the comparative sample performing a PCA by using a set of 28 anatomical landmarks. In the PCA we used the covariance matrix built on the comparative sample. Subsequently, we performed an ordinary Procrustes analysis to register the shapes of the three virtual reconstructions of Altamura on the mean shape of the comparative sample. Lastly, the registered landmark configurations of Altamura (based on the three models) were projected to predict its PC scores values.

### Cranial capacity

The endocranial volume of Altamura was estimated by warping the cranial endocast of SH-5, which was chosen as reference because it shows the lowest value of Procrustes distance from Altamura (Supplementary Data Fig. 4). We defined 29 homologous anatomical landmarks on the crania of SH-5 and Altamura. Of these, 28 out of 29 could be directly recorded on Altamura, whereas the only missing one (bregma) was estimated from SH-5 after Procrustes registration (shape and size). In addition, we defined 40 surface semilandmarks on Altamura (Supplementary Data Fig. 6) and we performed a projection and sliding procedure to find their correspondent position on the target SH-5 specimen. We applied *endomaker*^43^ to carry out the automatic segmentation of the SH-5 cranial endocast. Eventually, we warped the SH-5 endocast by TPS (function *tps3d* of the Morpho R package^44^), selecting the landmark and semilandmark configurations of Altamura as target. We estimated the cranial capacity of Altamura by calculating the volume of the warped endocast, which can be compared with data from literature^17,18,45–47^ (Supplementary Data Table 3).

### Discrete features

To evaluate the phylogenetic affinities of Altamura within the Neanderthal evolutionary lineage, we selected 20 non-metric features among those visible on the virtual reconstruction of the Altamura cranium. These non-metric features—related to splanchnocranium and neurocranium—are commonly used to define the clade of the so called ‘classic’ or European Würmian Neanderthals. The list of 20 characters is taken from Churchill (2014)^4^ comprehensive of the definition of each feature (Supplementary Data Table 4).

We compared Altamura with a sample comprising Middle Pleistocene European samples (MPH) (*N* = 18; Petralona and 17 individuals from Atapuerca Sima de Los Huesos), late Middle Pleistocene specimens (ERN) (*N* = 2; the crania from Saccopastore, Rome), and European Würmian Neanderthals (EWN) (*N* = 4). The specimens were selected according to their degree of completeness. Unfortunately, some important specimens are too fragmentary (e.g., the Krapina sample) or lacking large anatomical portions of basicranium and neurocranium (e.g., Arago, Apidima 2) to be included in the phylogenetic analysis. The sampling of non-metric features was carried out by two of us (GM and FdV) on the 3D stereolithographic model of the Altamura cranium, on the original specimens of Saccopastore 1 and 2 and on museum quality casts of Petralona, SH-4, SH-5, La Ferrassie 1, La Chapelle-aux-Saints, La Quina 5, Guattari 1. Observations about the state of the characters within the larger sample of Atapuerca Sima de los Huesos are from literature^6^. We defined the character status of the Saccopastore sample by combining the scores of Saccopastore 1 and Saccopastore 2. For Atapuerca SH sample (*N* = 17), a majority criterion was adopted according to the data reported in supplementary Table 6 from Arsuaga and colleagues^6^.

The nexus data matrix was processed with PAUP version 4.0b10^48^ using the Maximum Parsimony and the Neighbour-joining algorithms. We limited our analysis to features visible on the 3D reconstruction of the Altamura cranium; however, the score of five of these was assigned only tentatively, because overlying concretions may have altered or partially obscured the original morphology. Three of these five uncertain features were assigned to the “Neanderthal condition”. These are:

(i) position of the external auditory meatus on the same horizontal plane as the temporal zygomatic process (feature n. 17, Supplementary Data Table 4); this feature is typical of EWNs, whereas it is not fully expressed in both Saccopastore specimens. It is worth mentioning that only the left meatus is visible in Altamura (the right one is covered by speleothems and cannot be acquired digitally), as well as part of the zygomatic bar of the same side;
(ii) shape and depth of the mandibular fossa (feature n. 18, Supplementary Data Table 4); the fossa is preserved only on the left side and is partially filled with concretions;
(iii) shape and position of the *foramen magnum* (feature n. 19, Supplementary Data Table 4). Also in this case the sample of Saccopastore looks less derived than Altamura, which resembles shape and position of the *foramen magnum* in Saccopastore 1.

### Contour polylines describing the occipital bone morphology

The human occipital bone includes several diagnostic features commonly used to differentiate Neanderthals from Upper Palaeolithic modern humans and Middle to Late Pleistocene fossil hominins. We investigated in detail the 3D topology of the Altamura occipital bone by applying a method based on contour polylines, described by Boissonnat and colleagues^49^. This method maps the external morphological features of Altamura and of a sample of Middle to Late Pleistocene hominins used for comparison (Supplementary Data Fig. 7). The method is based on building closed polygonal chains lying on equidistant parallel planes cutting the mesh. The planes are set perpendicular to the axis defined by the maximum cranial length, calculated as glabella-opistocranion chord. In this way, a number of equidistant contour curves (polylines) are drawn on the cranium. We used the morphology of this set of curves to highlight the anatomical features of Altamura. Polylines were digitally defined using Mimics (Materialise NV, Leuven, Belgium) on each specimen by calculating a series of non-overlapping closed polygons spaced by 2.0 mm. We used SH-5 from Atapuerca for reference in aligning the mid-sagittal planes (glabella, bregma and lambda) of all specimens. Finally, the evenly-spaced polylines were automatically generated starting from the opistocranion and the topology of the occipital squama was returned.

### Shape analysis of the occipital bone

The occipital bone of Altamura is reasonably free from calcite coatings, so that a detailed study of its morphology by Geometric Morphometric methods can be carried out. We performed two different analyses: one on the entire occipital squama, the other on the midsagittal profile, from the lambda to the middle point between the inferior nuchal lines (Fig. 4). We captured the occipital bone morphology by defining 5 landmarks and 150 surface semi-landmarks on the squama (Fig. 4, Supplementary Data Table 2). Then, we projected the set of surface semilandmarks on 50 specimens of a reference sample (Supplementary Data Table 1) including *Homo sapiens* (*N* = 41), *Homo neanderthalensis* (*N* = 7) and Middle to Late Pleistocene human fossil specimens (*N* = 3). In addition, we compared the morphology of the Altamura occipital (Fig. 4a) with the mean shapes of Neanderthals (Fig. 4b), Middle Pleistocene humans (Fig. 4c) and modern humans (Fig. 4d). The semilandmark configurations on Altamura and the mean shapes of the subsamples were converted into triangular meshes. We display the differences between Altamura and the three comparative groups as local variation (contraction and expansion), by converting the area of each facet into a colour map using the function *localmeshdiff* embedded in the Arothron R package^50^. All analyses were performed in R environment^51^.

### Reporting summary

Further information on research design is available in the Nature Portfolio Reporting Summary linked to this article.

## Supplementary information

Supplementary Material

Reporting Summary

## Data Availability

All data needed to evaluate the conclusions of the paper are present in the article and Supplementary Data. Data and R code are available at 10.6084/m9.figshare.21900495.
