# Supplementary material for: Virtual excavation and analysis of the early Neanderthal cranium from Altamura (Italy)
Source: Commun Biol. 2023 Mar 24;6:316. doi: 10.1038/s42003-023-04644-1 (PMC10039001; doi:10.1038/s42003-023-04644-1)
Supplement: Supplementary file 1 — Supplementary Material [file 42003_2023_4644_MOESM1_ESM.pdf]

# Virtual excavation and analysis of the early Neanderthal cranium from Altamura (Italy)

Antonio Profico, Costantino Buzi, Fabio Di Vincenzo, Marco Boggioni, Andrea Borsato, Giovanni Boschian, Damiano Marchi, Mario Micheli, Jacopo Moggi Cecchi, Marco Samadelli, Mary Anne Tafuri, Juan Luis Arsuaga & Giorgio Manzi

***SUPPLEMENTARY DATA***

*Suppl. Figures 1-14* ... ... ... ... ... ... ... ... ... *pag. 2-15*

*Suppl. Tables 1-4* ... ... ... ... ... ... ... ... ... *pag. 16-24*

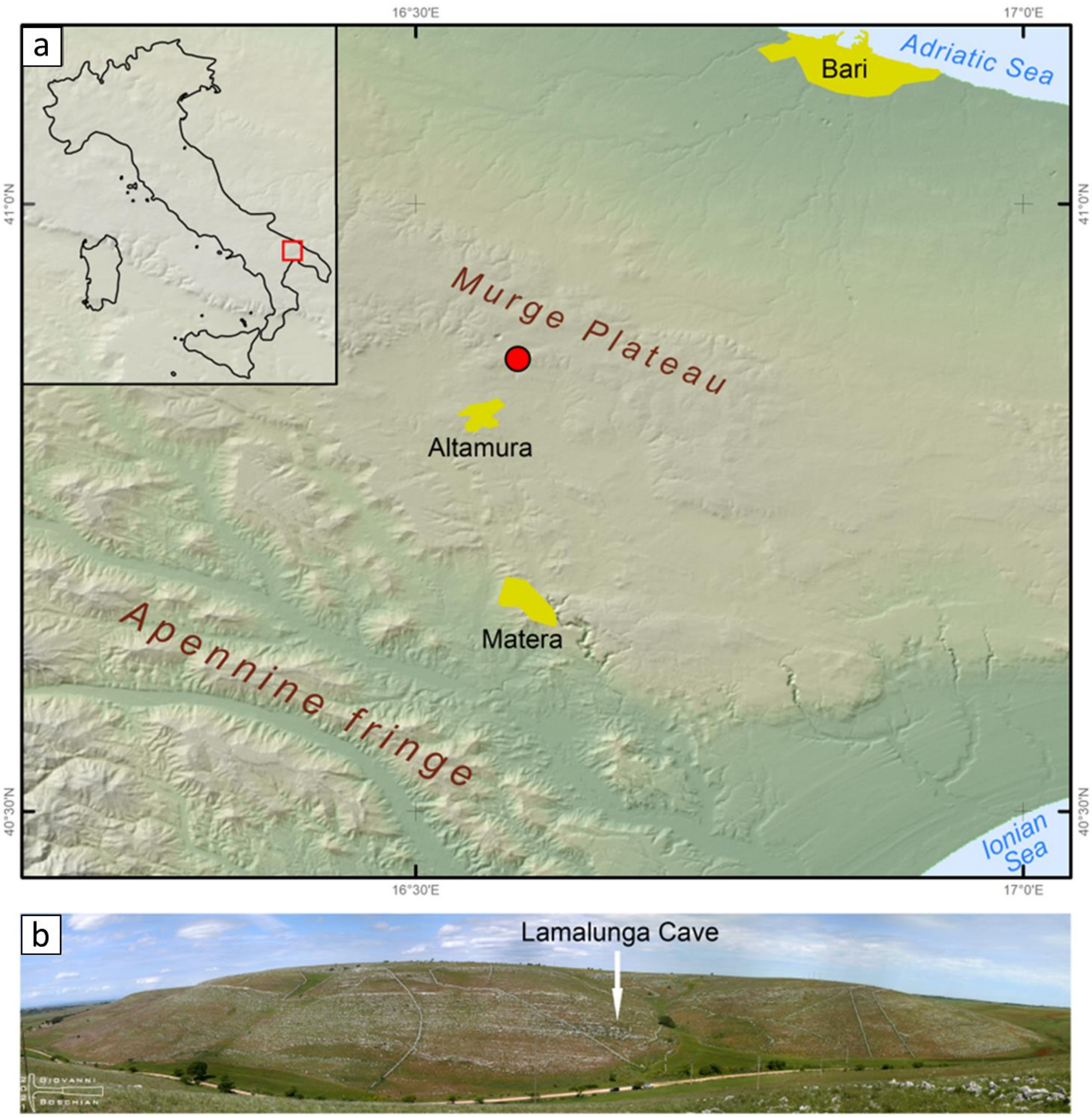


**Supplementary Figure 1.** Location map of Altamura within the Murge karstic plateau in South-Eastern Italy, between Apulia and Lucania regions. **a**: The Murge plateau (map center) is an extensive karstic area, characterised by typical karst landforms and numerous caves. The Lamalunga cave, where the Altamura human remains were found, is indicated by a red dot. **b**: Typical Murge Plateau landscape around Lamalunga cave entrance (arrow), showing the "lama", i.e. a shallow valley-shaped landforms, and the layering of limestone strata. Image credits: a) terrain relief sourced from 1-ArcSec Shuttle Radar Topography Mission elevation available from the U.S. Geological Survey (https://www.usgs.gov/centers/eros/science/usgs-eros-archive-digital-elevation-shuttle-radar-topography-mission-srtm-1) and processed with QGIS 3.28 Firenze (https://qgis.org/), graphic elaboration by GB; b) image by GB.

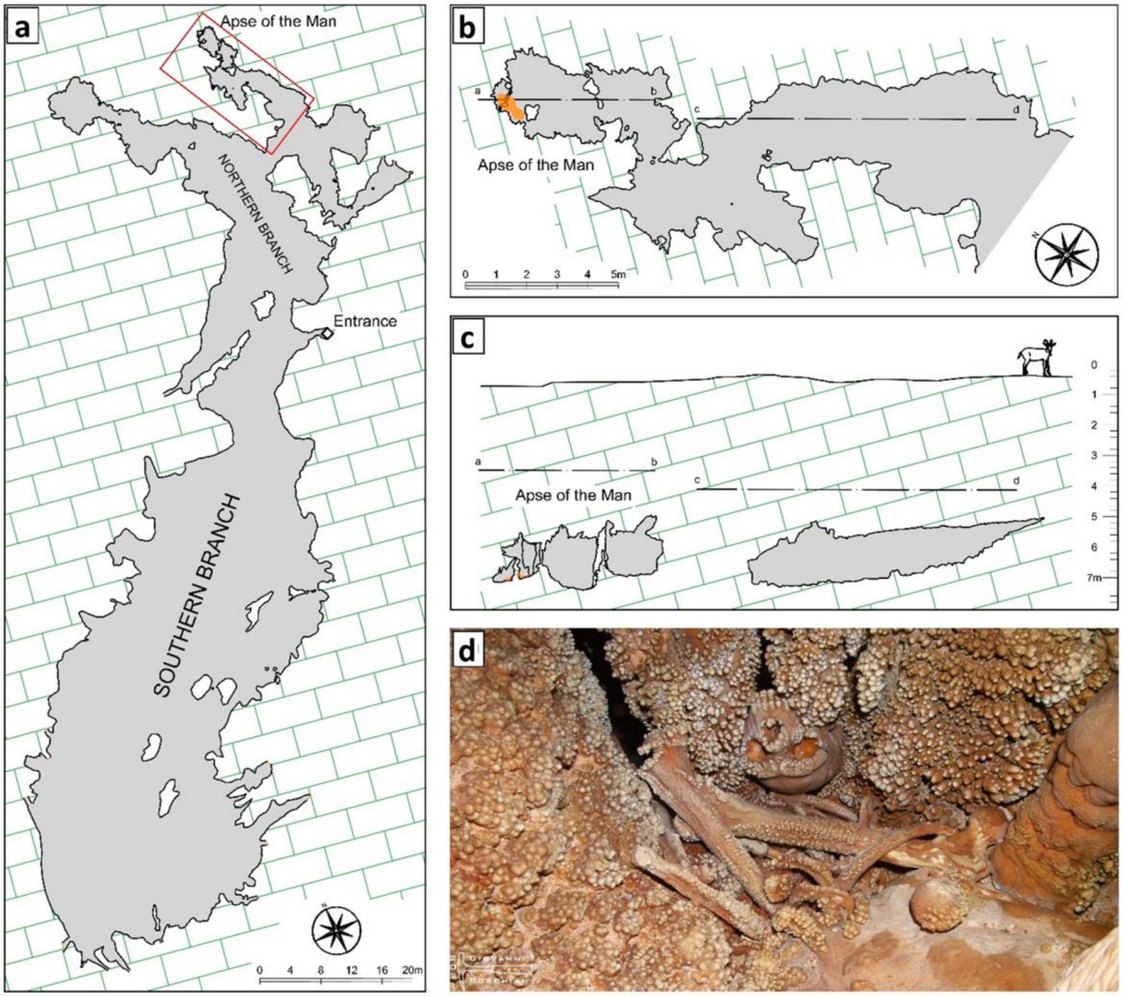


**Supplementary Figure 2.** Lamalunga cave simplified plan, showing the location of human remains. **a**: the karstic system of Lamalunga is divided into the Southern and Northern branches, with the present-day artificial entrance (Eastern side) located between the two branches. The red rectangle highlights the detail (**b**) of the Northernmost part of the cave and of the "Apse of the Man", with its narrow passages and location of the human remains (amber shading). **c**: the profiles of the northernmost part of the cave (a-b and c-d tracks) show the relatively shallow position of the human remains (amber shading). **d**: the Altamura cranium is cemented among columnar stalactites/stalagmites and covered with coralloid concretions. Several long bones lie in front of the cranium. Image credits: a, b, c) panels simplified and redrawn from Ministero della Cultura, Soprintendenza A.B.A.P. per la c.m. di Bari (Puglia); d) Soprintendenza A.B.A.P. per la c.m. di Bari (Puglia), photo by GB.

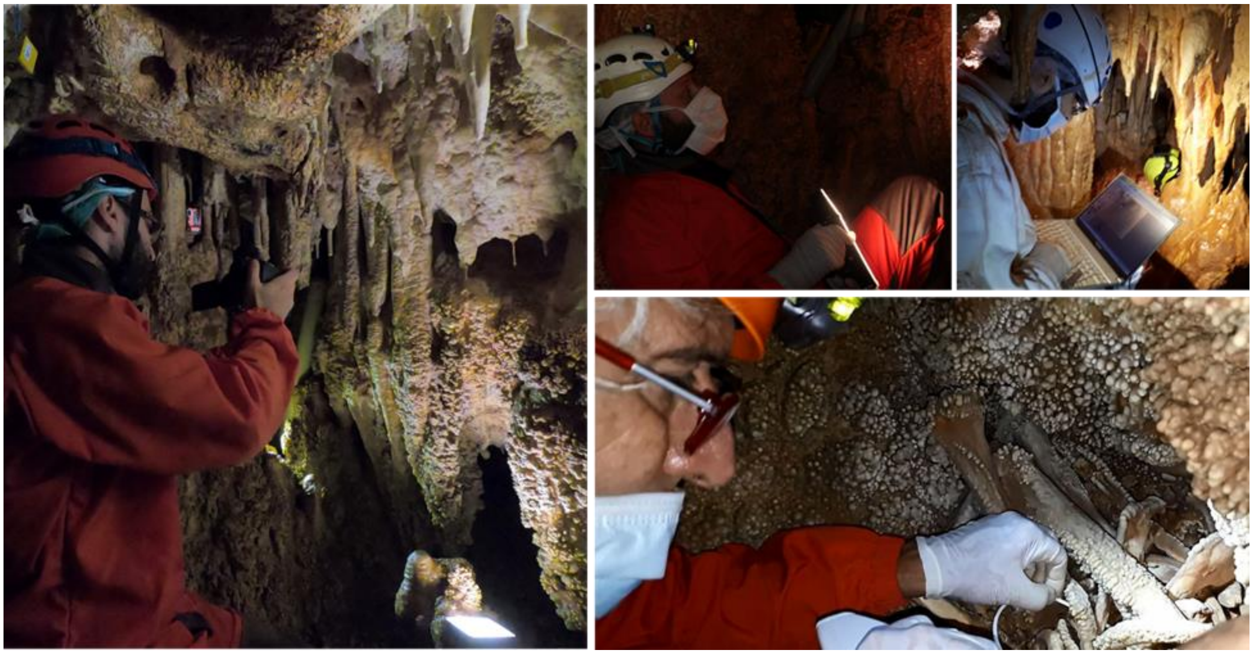

**Supplementary Figure 3.** Some of the activities carried out in the Apse and on the bone assemblage. Clockwise: i) Photographic documentation of speleothems in the Apse; ii) positioning of LED lights for acquisition of images of the bone assemblage by DinoLite digital microscope; iii) exploration of the assemblage by endoscopic probe (photos by AP and CB).

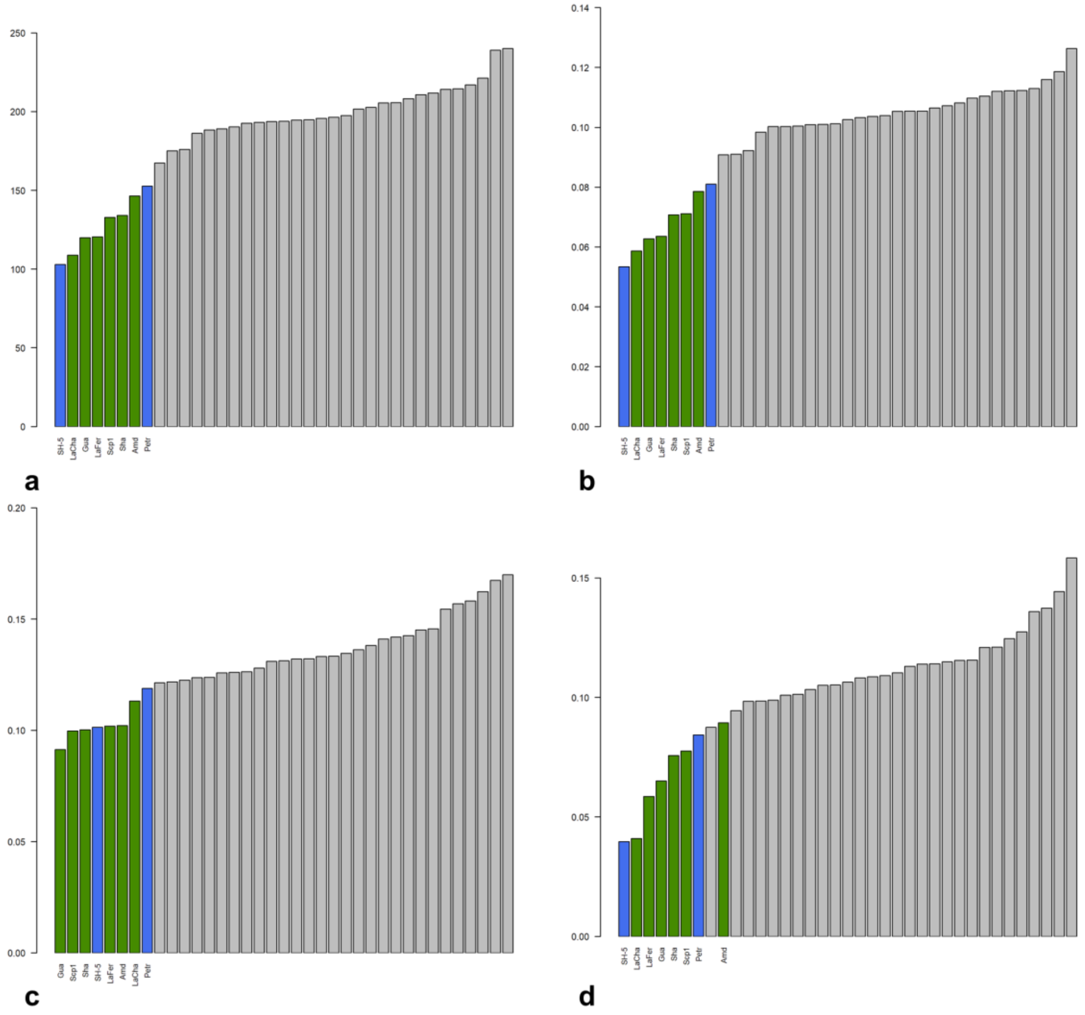


**Supplementary Figure 4.** Barplot of Euclidean (a) and Procrustes (b) distances between Altamura and each specimen of the comparative sample; barplots of the Procrustes distance calculated separately on FF (c) and BP (d); blue: Middle Pleistocene humans; green: Neanderthals; grey: *Homo sapiens*. SH-5: Sima de los Huesos 5; LaCha: La Chapelle; LaFer: La Ferrassie; Gua: Guattari 1; Scp1: Saccopastore 1; Sha: Shanidar; Amd: Amud; Petr: Petralona

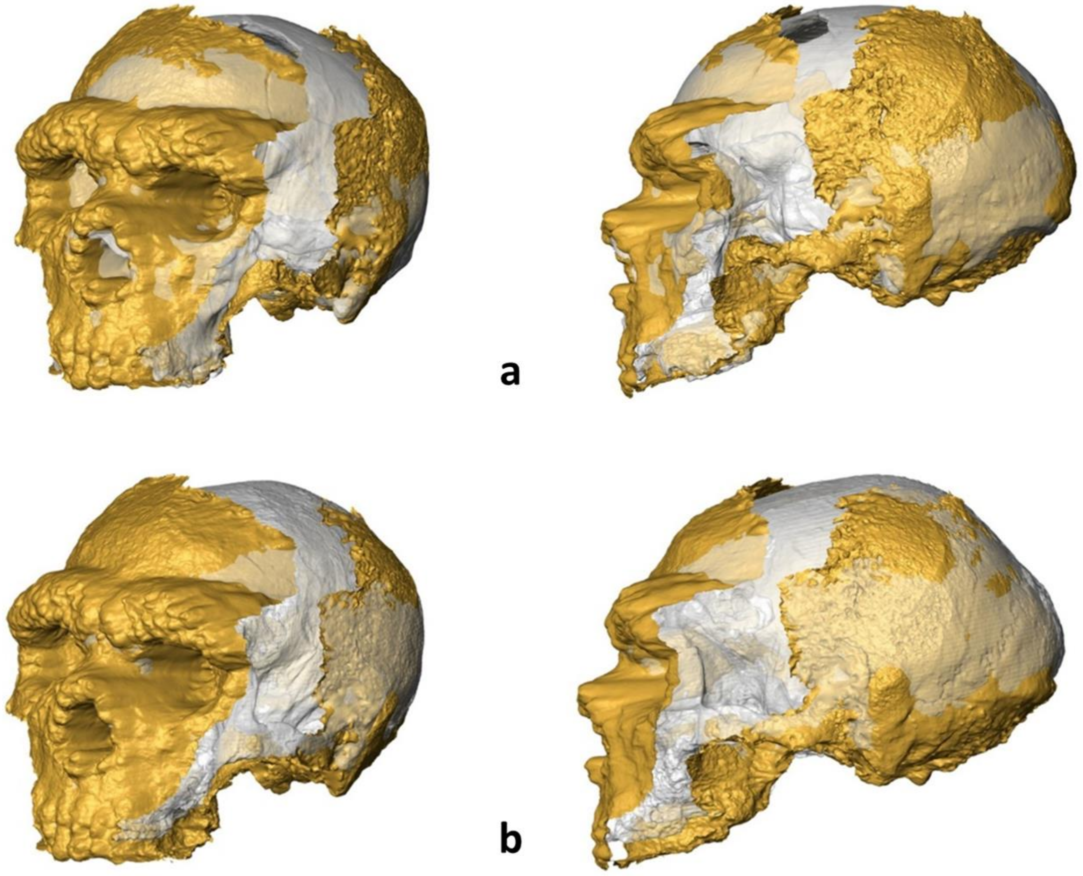


**Supplementary Figure 5.** Three-quarters left (left) and lateral left views (right) of virtually assembled models of the two separately digitised parts (front and face [FF] and cranial base and cranial vault [BP]) of the Altamura cranium, obtained by applying DTA software. Reference models used: **a**) Saccopastore 1; **b**) consensus shape of EWN sample.

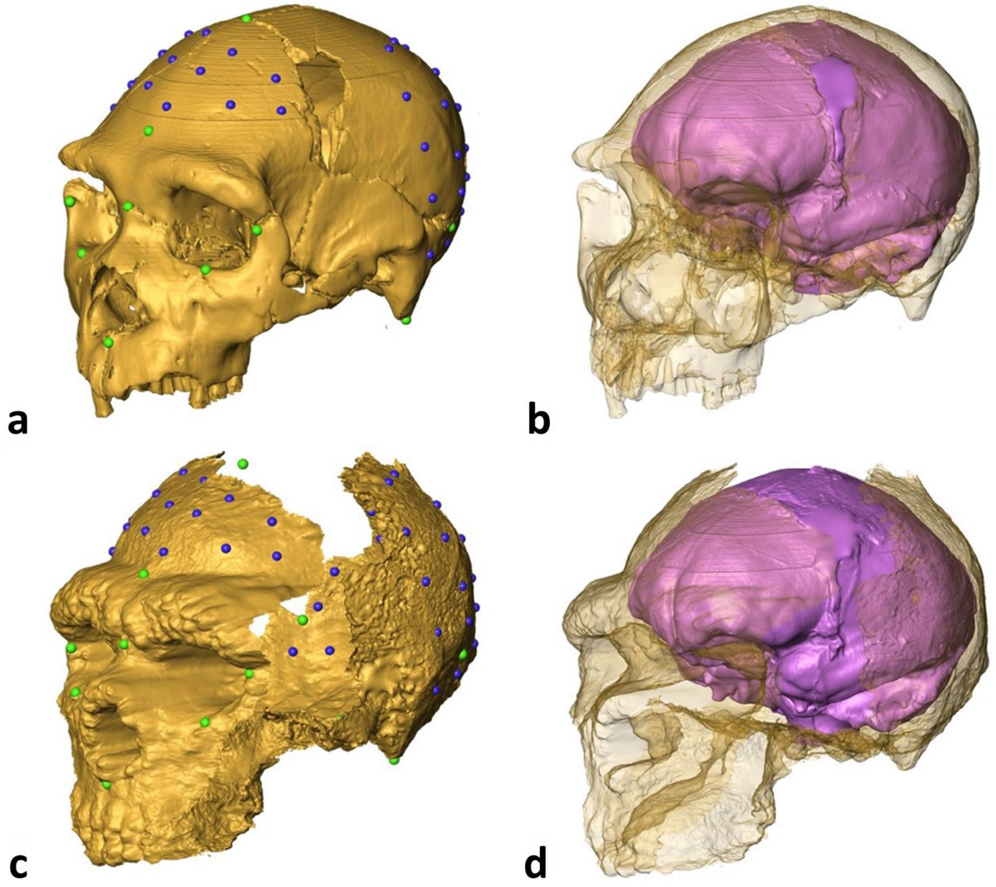


**Supplementary Figure 6.** Specimen SH-5 from Atapuerca (**a**) was used as reference to estimate cranial capacity in Altamura. The SH-5 endocast (**b**) was warped by using the same landmarks (green) and semilandmark (blue) configuration acquired on Altamura (**c**). The endocast warped on Altamura was used to estimate its cranial capacity (**d**).

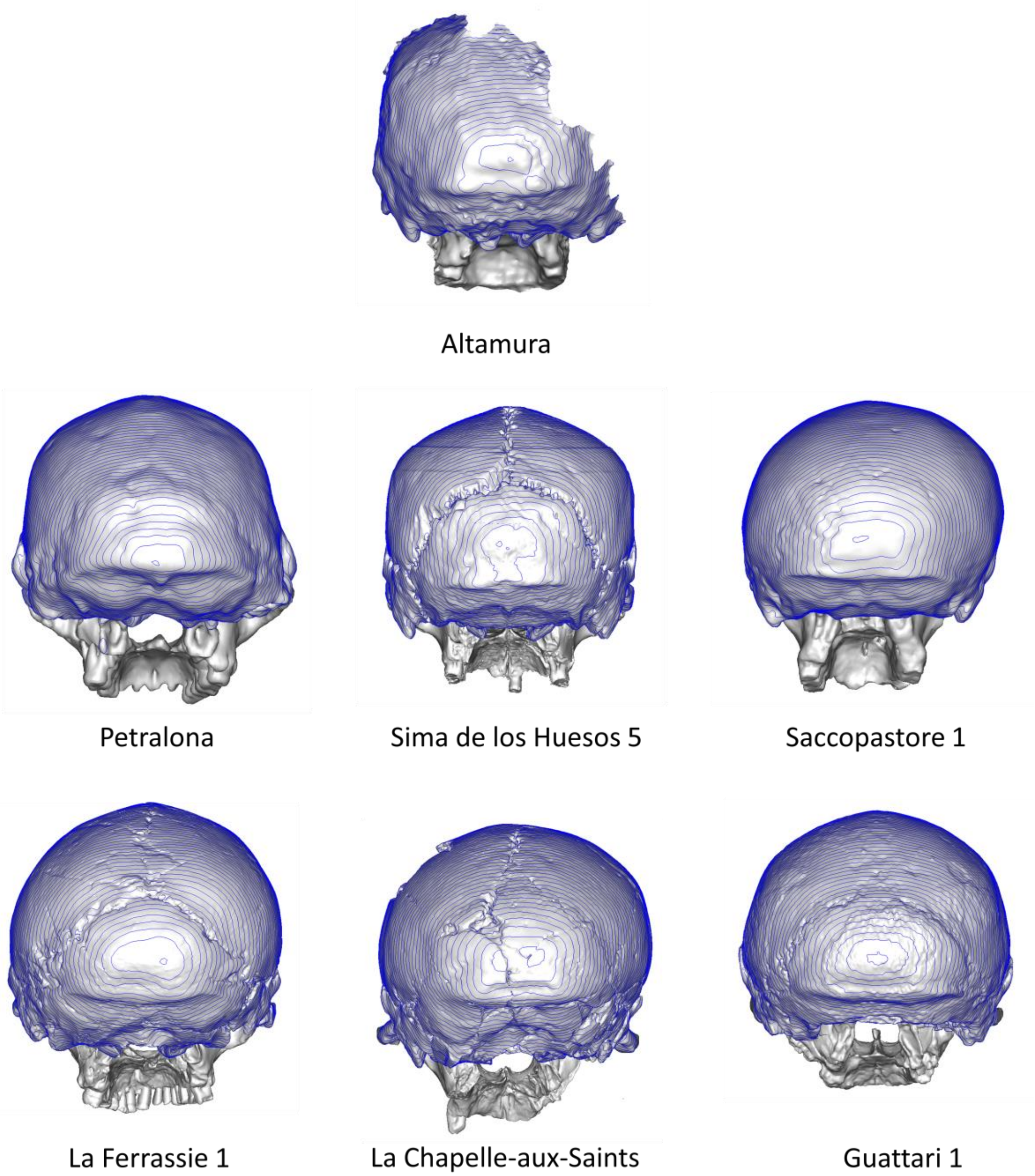


**Supplementary Figure 7.** 3D contour polylines (blue) traced on the occipital region of Altamura and of selected Middle to Late Pleistocene human fossils.

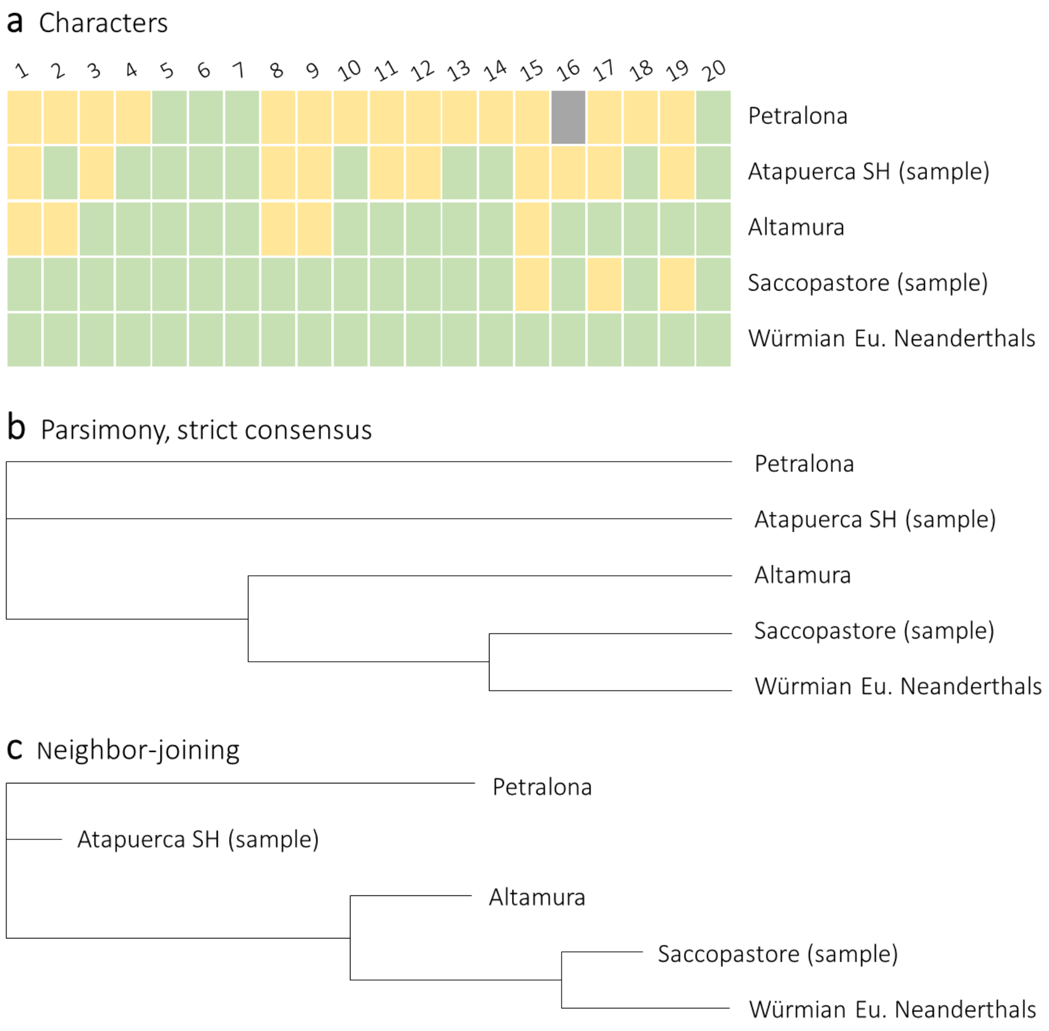


**Supplementary Figure 8.** Discrete features in Altamura. **a**: occurrence table of 20 synapomorphic Neanderthal traits on Altamura and on each sample, as defined by Churchill[4]; green: Neanderthal character state; yellow: non-Neanderthal character state; grey: data not available. These data were used to perform two different clustering methods, **b**: "parsimony, strict consensus" and **c:** "neighbour-joining".

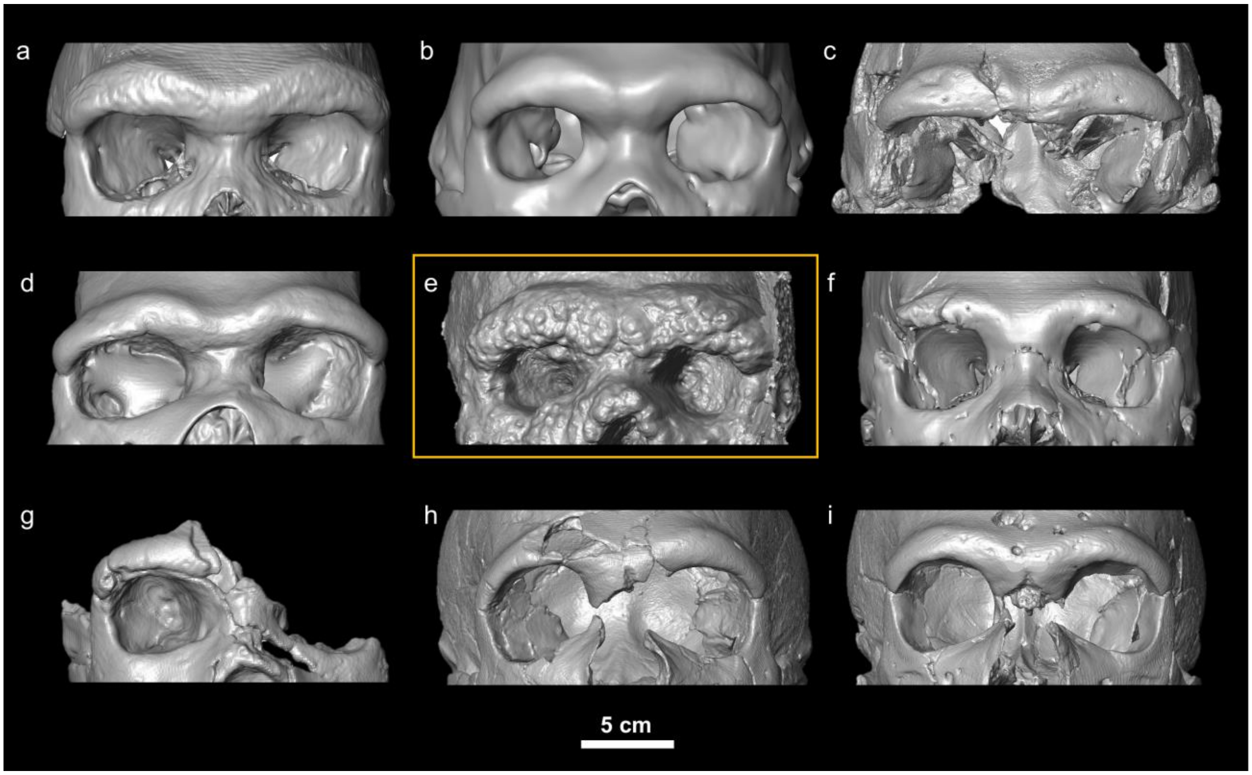


**Supplementary Figure 9.** Morphology of the supraorbital torus in Altamura compared to Middle Pleistocene humans and Neanderthals: a) Kabwe (or Broken Hill 1); b) Petralona; c) Ceprano; d) Arago XXI; e) *Altamura* (orange box); f) Sima de los Huesos 5; g) Saccopastore 2; h) La Ferrassie 1; i) La Chapelle-aux-Saints 1.

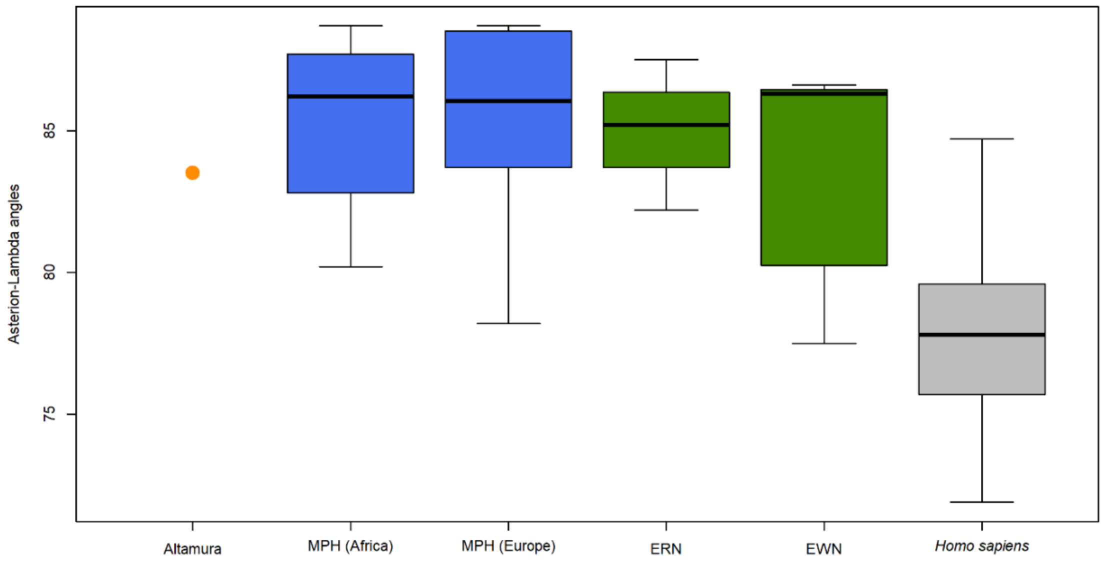


**Supplementary Figure 10.** Boxplot of the angle between the lambda and the two asteria in Altamura and reference sample. MPH: Middle Pleistocene Humans; ERN: early Neanderthals; EWN: European Würmian Neanderthals.

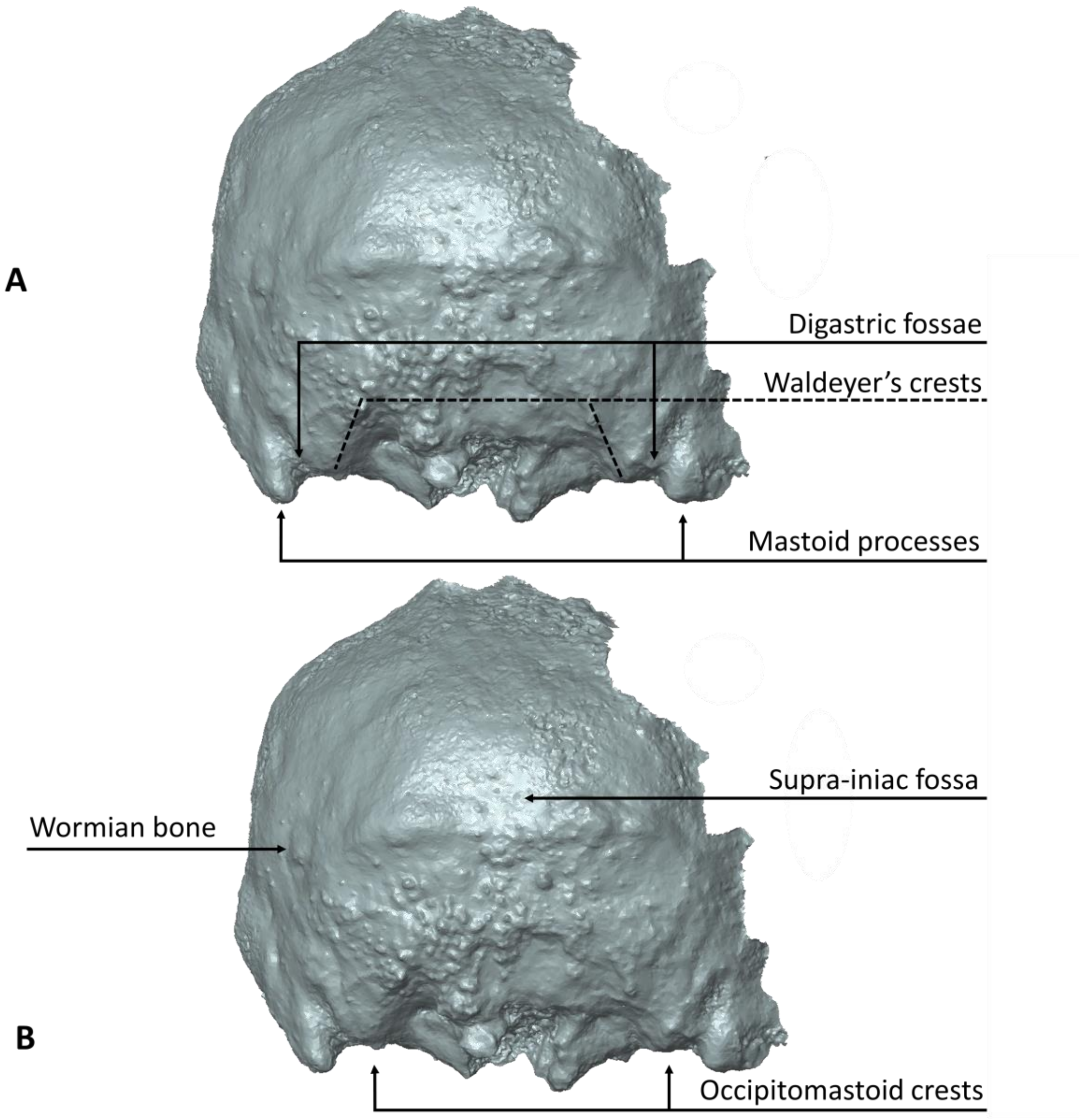


**Supplementary Figure 11.** Diagnostic anatomical features on the occipital bone of Altamura in posterior view (A, B).

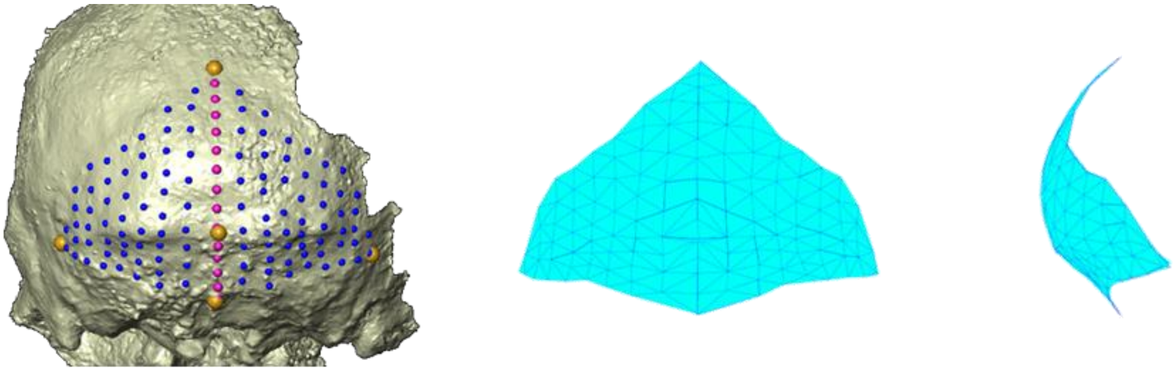

a b c

**Supplementary Figure 12.** Landmark set (yellow), surface semilandmarks (blue) and outline (fuchsia) used to study the occipital squama in Altamura (a). The triangulation of the entire landmark and semilandmark configuration, here reported in posterior (b) and lateral (c) view, has been used as a template to plot the shape variations.

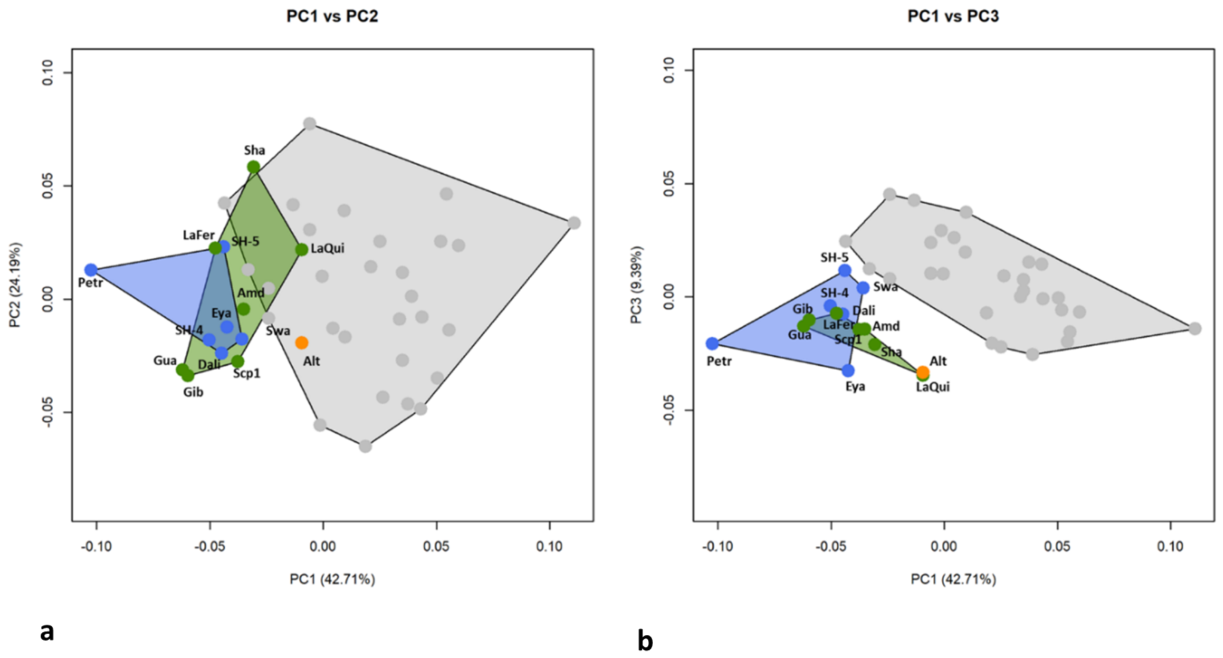


**Supplementary Figure 13.** Principal Component Analysis of landmarks set on the occipital squama (a: PC1 vs PC2; b: PC1 vs. PC3). Full circles represent fossil specimens; orange: Altamura (Alt); blue: Mid-Pleistocene specimens (SH-4: Sima de los Huesos 4; SH-5: Sima de los Huesos 5; Petr: Petralona; Eya: Eyasi; Swa: Swanscombe); green: *Homo neanderthalensis* (Amd: Amud; Gua: Guattari 1; LaFer: La Ferrassie; Scp1: Saccopastore 1; Sha: Shanidar; Gib: Gibraltar; LaQui: La Quina 5); grey: *Homo sapiens*.

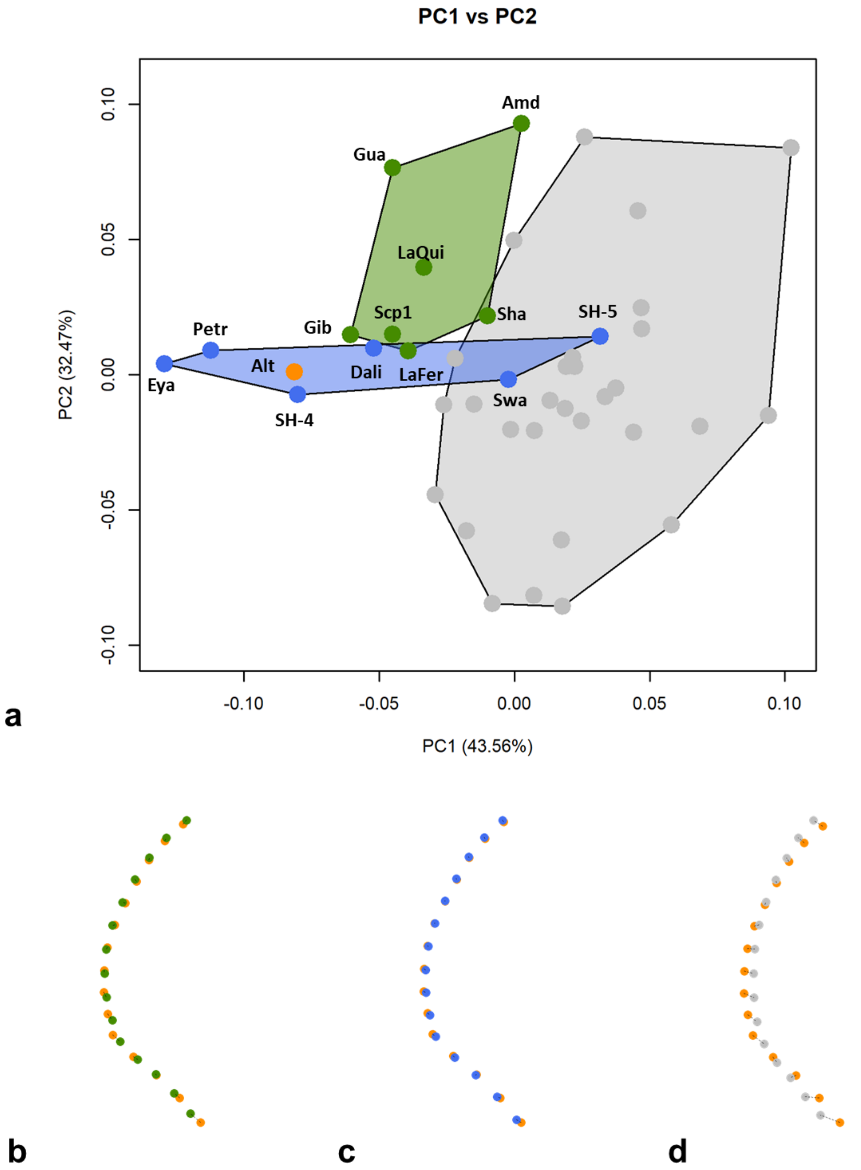


**Supplementary Figure 14.** Plot of the first two principal component derived from landmarks set on the occipital midsagittal profile (a). Full circles represent fossil specimens; orange: Altamura (Alt); blue: Mid-Pleistocene specimens (SH-4: Sima de los Huesos 4; SH-5: Sima de los Huesos 5; Petr: Petralona; Eya: Eyasi; Swa: Swanscombe); green: *Homo neanderthalensis* (Amd: Amud; Gua: Guattari 1; LaFer: La Ferrassie; Scp1: Saccopastore 1; Sha: Shanidar; Gib: Gibraltar; LaQui: La Quina 5); grey: *Homo sapiens*. In the bottom row, the shape variations of the first two principal components of Altamura (in orange) are compared to *Homo neanderthalensis* (green, b), Mid-Pleistocene humans (blue, c) and modern humans (grey, d).

**Supplementary Table 1.** List of the specimens used to digitally align (DTA) face and frontal bone (FF) with the base and posterior portions of the Altamura cranium (BP). The same list was also used in the morphometric analysis of the occipital bone (3D OCC GM). Fossil specimens' abbreviations are in brackets.

| Specimen | Species/taxon | Analysis |
|---|---|---|
| **Petralona (Petr)** | Middle Pleistocene Humans | DTA, 3D OCC GM |
| **Dali** | Middle Pleistocene Humans | 3D OCC GM |
| **Eyasi (Eya)** | Middle Pleistocene Humans | 3D OCC GM |
| **Sima de los Huesos 5 (SH-5)** | Middle Pleistocene Humans | DTA, 3D OCC GM |
| **Sima de los Huesos 4 (SH-4)** | Middle Pleistocene Humans | 3D OCC GM |
| **Swanscombe (Swa)** | Middle Pleistocene Humans | 3D OCC |
| **Amud 1 (Amd)** | *Homo neanderthalensis* | DTA, 3D OCC GM |
| **Saccopastore 1 (Scp1)** | *Homo neanderthalensis* | DTA, 3D OCC GM |
| **Guattari 1 (Gua)** | *Homo neanderthalensis* | DTA, 3D OCC GM |
| **La Chapelle aux Saints 1 (LaCha)** | *Homo neanderthalensis* | DTA |
| **La Ferrassie 1 (LaFer)** | *Homo neanderthalensis* | DTA, 3D OCC GM |
| **La Quina 5 (LaQui)** | *Homo neanderthalensis* | 3D OCC GM |
| **Gibraltar 1 (Gib)** | *Homo neanderthalensis* | 3D OCC GM |
| **Shanidar 1 (Sha)** | *Homo neanderthalensis* | DTA, 3D OCC GM |
| **OL 0794** | *Homo sapiens* | DTA, 3D OCC GM |
| **OL 0869** | *Homo sapiens* | DTA, 3D OCC GM |
| **OL 0886** | *Homo sapiens* | 3D OCC GM |
| **OL 1068** | *Homo sapiens* | DTA, 3D OCC GM |
| **OL 1112** | *Homo sapiens* | DTA, 3D OCC GM |
| **OL 1193** | *Homo sapiens* | 3D OCC GM |

| | | |
|---|---|---|
| **OL 1197** | *Homo sapiens* | DTA, 3D OCC GM |
| **OL 1199** | *Homo sapiens* | DTA, 3D OCC GM |
| **OL 1282** | *Homo sapiens* | 3D OCC GM |
| **OL 1214** | *Homo sapiens* | 3D OCC GM |
| **OL 1282** | *Homo sapiens* | 3D OCC GM |
| **OL 1428** | *Homo sapiens* | 3D OCC GM |
| **ULAC 012** | *Homo sapiens* | DTA |
| **ULAC 013** | *Homo sapiens* | DTA |
| **ULAC 016** | *Homo sapiens* | DTA |
| **ULAC 019** | *Homo sapiens* | DTA |
| **ULAC 033** | *Homo sapiens* | DTA |
| **ULAC 039** | *Homo sapiens* | DTA |
| **ULAC 057** | *Homo sapiens* | DTA |
| **ULAC 060** | *Homo sapiens* | DTA |
| **ULAC 066** | *Homo sapiens* | DTA |
| **ULAC 904** | *Homo sapiens* | DTA |
| **ULAC 909** | *Homo sapiens* | DTA |
| **ULAC 920** | *Homo sapiens* | DTA |
| **ULAC 953** | *Homo sapiens* | DTA |
| **ULAC 954** | *Homo sapiens* | DTA |
| **ULAC 955** | *Homo sapiens* | DTA |
| **VA-004** | *Homo sapiens* | 3D OCC GM |
| **VA-005** | *Homo sapiens* | 3D OCC GM |
| **VA-011** | *Homo sapiens* | 3D OCC GM |

| | | |
|---|---|---|
| **VA-012** | *Homo sapiens* | DTA |
| **VA-013** | *Homo sapiens* | 3D OCC GM |
| **VA-014** | *Homo sapiens* | 3D OCC GM |
| **VA-017** | *Homo sapiens* | 3D OCC GM |
| **VA-018** | *Homo sapiens* | 3D OCC GM |
| **VA-019** | *Homo sapiens* | 3D OCC GM |
| **VA-020** | *Homo sapiens* | 3D OCC GM |
| **VA-021** | *Homo sapiens* | DTA, 3D OCC GM |
| **VA-022** | *Homo sapiens* | 3D OCC GM |
| **VA-023** | *Homo sapiens* | 3D OCC GM |
| **VA-024** | *Homo sapiens* | 3D OCC GM |
| **VA-027** | *Homo sapiens* | 3D OCC GM |
| **VA-029** | *Homo sapiens* | DTA |
| **VA-030** | *Homo sapiens* | DTA, 3D OCC GM |
| **VA-031** | *Homo sapiens* | DTA |
| **VA-032** | *Homo sapiens* | DTA, 3D OCC GM |
| **VA-033** | *Homo sapiens* | DTA, 3D OCC GM |

**Supplementary Table 2.** List of landmarks used in digitally aligning the two cranial portions of Altamura (DTA), as well as in the morphometric analysis of the occipital bone (3D OCC GM). *Legend:* "Pairing" indicates the position of the landmarks; MS: mid-sagittal plane; BL: bilateral (both sides); "Portion" indicates the separately acquired part of the cranium where the landmark is located; FF: face and frontal bone; BP: base and posterior portions of the cranium.

| ***Landmark name/description*** | ***Pairing*** | ***Portion*** | ***Analysis*** |
|---|---|---|---|
| *Supra-glabellar depression (medial point)* | MS | FF | DTA |
| *Nasion* | MS | FF | DTA |
| *Nasospinale* | MS | FF | DTA |
| *Prosthion* | MS | FF | DTA |
| *Supraorbital notch* | BL | FF | DTA |
| *Frontomalare orbitale* | BL | FF | DTA |
| *Zygoorbitale* | BL | FF | DTA |
| *Lacrimale* | BL | FF | DTA |
| *Alare* | BL | FF | DTA |
| *Infraorbital foramen* | BL | FF | DTA |
| *Lambda* | MS | BP | DTA, 3D OCC GM |
| *Inion* | MS | BP | DTA, 3D OCC GM |
| *Inferior nuchal lines (middle point)* | MS | BP | 3D OCC GM |
| *Basion* | MS | BP | DTA |
| *Hormion* | MS | BP | DTA |
| *Posterior margin of the palatine foramen* | MS | BP | DTA |
| *Orale* | MS | BP | DTA |
| *Asterion* | BL | BP | DTA, 3D OCC GM |
| *Mastoidale* | BL | BP | DTA |
| *Posteriormost point of the maxillary dental arch* | BL | BP | DTA |

**Supplementary Table 3.** Comparative cranial capacity in selected Pleistocene fossil humans. Values in bold were calculated on original specimens by the authors, while those in regular font are reported from literature[17,18,45-47], including some estimates for partial specimens.

| **Species/group** | **Specimen** | **Endocranial capacity (cm$^3$)** |
|---|---|---|
| *MPH (Africa)* | Broken Hill | 1280[45] |
| *MPH (Africa)* | Bodo | 1250[45] |
| *MPH (Africa)* | Jebel Irhoud 1 | 1305[18] |
| *MPH (Africa)* | Laetoli 18 | 1367[18] |
| *MPH (Africa)* | Ndutu | 1100[45] |
| *MPH (Africa)* | Omo-Kibish 2 | 1435[18] |
| *MPH (Africa)* | Saldanha | 1200[45] |
| *MPH (Europe)* | Ceprano | **1150** |
| *MPH (Europe)* | Petralona | 1230[18] |
| *MPH (Europe)* | SH cranium 4 | 1390[18] |
| *MPH (Europe)* | SH cranium 5 | 1125[18] |
| *MPH (Europe)* | SH cranium 6 | 1140[45] |
| *MPH (Europe)* | Swanscombe | 1325[18] |
| *MPH (Europe)* | Steinheim | 1200[18] |
| *MPH (Europe)* | Arago XXI | 1166[45] |
| *MPH (Europe)* | Biache | 1200[45] |
| *MPH (Europe)* | Ehringsdorf | 1450[45] |
| *MPH (Europe)* | Reilingen | 1430[45] |
| *Homo neanderthalensis* | Apidima 2 | 1290[17] |
| *Homo neanderthalensis* | Ganovce | 1320[45] |
| *Homo neanderthalensis* | Krapina 1 | 1419[47] |

| | | |
|---|---|---|
| *Homo neanderthalensis* | Krapina 2 | 1286[47] |
| *Homo neanderthalensis* | Krapina 3 | 1272[47] |
| *Homo neanderthalensis* | Krapina 5 | 1397[47] |
| *Homo neanderthalensis* | Krapina 6 | 1158[45] |
| *Homo neanderthalensis* | Saccopastore 1 | **1103** |
| *Homo neanderthalensis* | Saccopastore 2 | 1300[45] |
| *Homo neanderthalensis* | Tabun 1 | 1271[18] |
| *Homo neanderthalensis* | Amud | 1740[18] |
| *Homo neanderthalensis* | Feldhofer | 1525[45] |
| *Homo neanderthalensis* | Gibraltar 1 | 1270[45] |
| *Homo neanderthalensis* | Guattari 1 | **1380** |
| *Homo neanderthalensis* | La Chapelle-aux-Saints | 1625[18] |
| *Homo neanderthalensis* | La Ferrassie 1 | 1640[18] |
| *Homo neanderthalensis* | La Quina 5 | 1172[18] |
| *Homo neanderthalensis* | La Quina 18 | 1200[18] |
| *Homo neanderthalensis* | Le Moustier 1 | 1564[45] |
| *Homo neanderthalensis* | Shanidar 1 | 1600[45] |
| *Homo neanderthalensis* | Shanidar 5 | 1550[45] |
| *Homo neanderthalensis* | Spy 1 | 1305[18] |
| *Homo neanderthalensis* | Spy 2 | 1527[47] |
| *Homo neanderthalensis* | Theshik Tash 1 | 1550[45] |
| *Homo sapiens* | Skhul 5 | 1520[18] |
| *Homo sapiens* | Mladeč 1 | 1540[18] |
| *Homo sapiens* | CT-CSIC OL794 | **1280** |

| | | |
|---|---|---|
| *Homo sapiens* | CT-CSIC OL866 | **1433** |
| *Homo sapiens* | CT-CSIC OL869 | **1445** |
| *Homo sapiens* | CT-CSIC OL886 | **1276** |
| *Homo sapiens* | CT-CSIC OL1068 | **1248** |
| *Homo sapiens* | CT-CSIC OL112 | **1397** |
| *Homo sapiens* | CT-CSIC OL1187 | **1621** |
| *Homo sapiens* | CT-CSIC OL1192 | **1442** |
| *Homo sapiens* | CT-CSIC OL1193 | **1296** |
| *Homo sapiens* | CT-CSIC OL1197 | **1337** |
| *Homo sapiens* | CT-CSIC OL1199 | **1191** |
| *Homo sapiens* | CT-CSIC OL1214 | **1235** |
| *Homo sapiens* | CT-CSIC OL1282 | **1548** |
| *Homo sapiens* | CT-CSIC OL1428 | **1192** |

**Supplementary Table 4.** List of non-metric traits used in the phenetic analysis (from Churchill[4]). *Legend*: 0 = absence; 1 = presence, NA = not detectable. EWN = European Würmian Neanderthals, PET = Petralona, SH = Sima de los Huesos sample, SAC = Saccopastore sample, ALT = Altamura.

| Definition | EWN | PET | SH | SAC | ALT |
|---|---|---|---|---|---|
| *1 - Double-arched supraorbital torus that is arcuately continuous across glabella and that thins laterally.* | 1 | 0 | 0 | 1 | 0 |
| *2 - No separation between the arcus superciliaris and arcus supraorbitalis.* | 1 | 0 | 1 | 1 | 0 |
| *3 - High, rounded orbits* | 1 | 0 | 0 | 1 | 1 |
| *4 - Mid-facial prognathism characterised by low nasiofrontal and subspinal angles and a large difference between M1 alveolus and zygomaxillare radii.* | 1 | 0 | 1 | 1 | 1 |
| *5 - Flat or convex infraorbital plates (no canine fossa) enclosing large maxillary sinuses and receding obliquely in alignment with the anterolaterally flattened surface of the zygomatic.* | 1 | 1 | 1 | 1 | 1 |
| *6 - Posterior rooting of the facial (zygomatico-alveolar) crest.* | 1 | 1 | 1 | 1 | 1 |
| *7 - Platycephalic cranial vault with a large endocranial capacity* | 1 | 1 | 0 | 1 | 1 |
| *8 - Cranial vault shape "en bombe" in norma occipitalis.* | 1 | 0 | 0 | 1 | 0 |
| *9 - Maximum cranial breadth situated low on the parietals and indistinct parietal bosses* | 1 | 0 | 0 | 1 | 0 |
| *10 - Horizontal occipital torus of uniform vertical thickness, limited to the central portion of the occipital* | 1 | 0 | 1 | 1 | 1 |
| *11 – Depression instead of external occipital protuberance.* | 1 | 0 | 0 | 1 | 1 |
| *12 – Bipartite occipital torus with its median portion weakly developed and marked bilateral extension.* | 1 | 0 | 0 | 1 | 1 |
| *13 - Well developed, horizontally extended suprainiac fossa, triangular in outline with the apex in the midline and the base in contact with the occipital torus, and* | 1 | 0 | 1 | 1 | 1 |

| | | | | | |
|---|---|---|---|---|---|
| *generally demarked by an uplifted area of bone surrounding the depression.* | | | | | |
| *14 - Convex occipital squama (planum occipitale).* | 1 | 0 | 1 | 1 | 1 |
| *15 - Occipitomastoid crest usually larger than the mastoid process, often straddling the occipitomastoid suture, separated from surrounding bone by distinct sulci medially and laterally.* | 1 | 0 | 0 | 0 | 0 |
| *16 - Laterally flattened and inferiorly tapering mastoid process with medially inclined apex.* | 1 | NA | 0 | 1 | 1 |
| *17 - External auditory meatus situated on the same horizontal plane as the temporal zygomatic process* | 1 | 0 | 0 | 0 | 1 |
| *18 - Mandibular (glenoid) fossa that are large, shallow and poorly delimited* | 1 | 0 | 1 | 1 | 1 |
| *19 - Anteroposteriorly long, mediolaterally narrow foramen magnum.* | 1 | 0 | 0 | 0 | 1 |
| *20 - Nasal (piriform) aperture that is tall and wide both inferiorly and superiorly (reflected in antero-inferiorly broad nasal bones).* | 1 | 1 | 1 | 1 | 1 |
